# Improved chickpea growth, physiology, nutrient assimilation and rhizoremediation of hydrocarbons by bacterial consortia

**DOI:** 10.1186/s12870-024-05709-x

**Published:** 2024-10-19

**Authors:** Muhammad Hayder Ali, Muhammad Imran Khan, Fiza Amjad, Naeem Khan, Mahmoud F. Seleiman

**Affiliations:** 1 Institute of Soil and Environmental Sciences, University of Agriculture, Faisalabad, 38000 Pakistan; 2 Agronomy Department, Institute of Food and Agricultural Sciences, University of Florida, Gainesville, FL 32611 USA; 3 Plant Production Department, College of Food and Agriculture Sciences, King Saud University, P.O. Box 2460, Riyadh, 11451 Saudi Arabia

**Keywords:** Phytoremediation, Petroleum hydrocarbons, Bacterial consortia, Chickpea, Rhizo-degradation

## Abstract

**Background:**

Soil pollution by petroleum hydrocarbons (PHCs) reduces yield by changing the physico-chemical properties of soil and plants due to PHCs’ biotoxicity and persistence. Thus, removing PHCs from the soil is crucial for ecological sustainability. Microbes-assisted phytoremediation is an economical and eco-friendly solution. The current work aimed to develop and use bacterial consortia (BC) for PHCs degradation and plant growth enhancement in hydrocarbon-contaminated soil. Initially, the enriched microbial cultures (that were prepared from PHCs-contaminated soils from five distinct regions) were obtained via screening through microcosm experiments. Afterward, two best microbial cultures were tested for PHCs degradation under various temperature and pH ranges. After culture optimization, isolation and characterization of bacterial strains were done to construct two BC. These constructed BC were tested in a pot experiment for hydrocarbons degradation and chickpea growth in PHCs contaminated soil.

**Results:**

Findings revealed that PHCs exerted significant phytotoxic effects on chickpea growth and physiology when cultivated in PHCs contaminated soil, reducing agronomic and physiological traits by 13–29% and 12–43%, respectively. However, in the presence of BC, the phytotoxic impacts of PHCs on chickpea plants were reduced, resulting in up to 24 − 35% improvement in agronomic and physiological characteristics as compared to un-inoculated contaminated controls. Furthermore, the bacterial consortia boosted chickpea’s nutritional absorption and antioxidant mechanism. Most importantly, chickpea plants phytoremediated 52% of the initial PHCs concentration; however, adding BC1 and BC2 with chickpea plants further increased this removal and remediated 74% and 80% of the initial PHCs concentration, respectively.

**Conclusion:**

In general, BC2 outperformed BC1 (with few exceptions) in promoting plant growth and PHCs elimination. Therefore, using multi-trait BC for PHCs degradation and plant growth improvement under PHCs stress may be an efficient and environmentally friendly strategy to deal with PHCs pollution and toxicity.

**Supplementary Information:**

The online version contains supplementary material available at 10.1186/s12870-024-05709-x.

## Introduction

Sustainable food supply for the world’s existing and upcoming population increases the food production demand on existing or degraded lands [1, 2]. Soil contamination with persistent organic pollutants especially the hydrocarbons (PHCs) and their derivatives is a major problem faced by the developing and developed world [3]. The PHCs become part of the soil by accidental spills, oil refineries, waste production, oil exploration and automobile workshops [4]. When these PHCs enter the soil systems, they change the physiochemical properties (like EC, pH, C:N ratio, porosity and water retention capacity) of the soil [5]. The plants grown in PHCs contaminated sites show stunted growth due to production of hydrogen peroxide (H_2_O_2_), ethylene and reactive oxygen species (ROS) [6]. These metabolites reduce the growth, yield and biomass production of plants by altering the nutrient intake and damaging the proteins, cell membrane and nucleic acid structures [7]. The introduction of PHCs in soil not only reduces plant growth but also decreases the native micro and macro biota [8].

There are several physicochemical methods to remediate the PHCs contents from the soil, but these methods require higher costs for technical labor, and in most cases, they produce harmful byproducts that are even more noxious than parent compounds [9]. However, the plants and microbes coupled bioremediation of PHCs is a suitable technique due to lower cost, more aesthetic value, and maximum chances of complete degradation of PHCs into CO_2_ and H_2_O [10]. Various microbes like fungi, algae and bacteria are widely used in the bioremediation of PHCs [11]. Among them, the bacterial communities are acting as a principal degrading agent as they use PHCs as a carbon (C) source for biomass production and reproduction [12, 13]. The bacterial isolates can break down the organic pollutants, but complete mineralization is very rare due to the unavailability of specific enzymes [14]. However, the microbial cultures with different bacterial strains can completely mineralize the PHCs as they increase the bioavailability and mobility of PHCs by exopolysaccharides secretions [15, 16]. This in turn increases the PHCs degradation in soil and eventually overcomes the phytotoxic impacts of PHCs on plant growth [17].

The PHCs removal in the rhizosphere could be enhanced by the proper bioaugmentation strategies [18]. Additionally, the supportive role of microbes through N fixation, siderophore production, phosphate solubilization, catalase, oxidase and ACC-deaminase activities further strengthens the plant microbe’s interaction even under stressful conditions [19]. The plant roots attract and support the nearby microbial communities by the secretion of root exudates which normally contain sugars, vitamins, organic acids, and amino acids [20]. Most root exudates have similar structures to low molecular weight PHCs fractions that’s why the microbial communities utilize them as a C substrate to improve their own growth and degrade the majority of the hydrocarbons in soil [21]. Moreover, the interactive effect of microbes and plants together could be beneficial for better physiology and improved plant growth and yield (i.e., plant height, biomass, number of leaves and grains yield) in PHCs-contaminated soil [7, 22].

Chickpea (*Cicer arietinum* L.) has been widely tested by scientists as a suitable candidate for the reclamation of contaminated sites [23, 24]. The plant has a larger and more extensive root system that improves the aeration and stabilization of soil structure and supports the biodegradation of pollutants [25]. The deeper root system enhances the bioavailability of hydrocarbons to several PHCs degrading microbes [26]. Most importantly, the plants can develop symbiotic relationships with N-fixing microbes and in turn improve soil fertility [27]. The exudation of various enzymes and organic acids attracts nearby microbiota, changes the rhizosphere pH and favorably improves soil health by maintaining the nutrient balance for plant growth even under contamination [28]. In leguminous crops, the degradation and accumulation of PHCs are mainly occurred in roots rather than the upper plant parts [29, 30]. However, the PHCs accumulation in leaves and grains is mostly dependent on aerial deposition rather than through root zone [31]. It has been well documented that leguminous crops favor the microbial communities that helps in enhancing PHCs breakdown in rhizosphere [32]. Additionally, most of PHCs have a higher octanol-water partition coefficient (logKow) which is more than three, tend to bound to the root surface and as a result the intake of contaminant in plants is reduced [30, 33]. However, there are still limited studies on hydrocarbon degrading indigenous bacterial consortia to combat hydrocarbon pollution and promote chickpea plant growth under pollutant stress.

In general, it is equally crucial to produce high-quality food and to eliminate or reduce the pollutant levels from the same soil. One most effective and environmentally appropriate methods to remove pollutants is to use microbes (especially the mixed bacterial consortia) and plants together [30]. The primary goal of this study was to evaluate the phytotoxicity of PHCs on chickpea plants and to find out how newly constructed bacterial consortia prove beneficial for the degradation of PHCs along with the development and growth of chickpea plants in soil contaminated with PHCs.

## Materials and methods

### Collection of soil samples

Initially, several PHCs contaminated sites were visited, and the soil samples were taken from the hotspots of the selected regions/sites across Pakistan including Rawalpindi (33°58’15.4” N, 73°15’12.9” E), Abbottabad (34°11’59.7” N, 73°14’29.9” E), Faisalabad (31°25’41.5” N 73°05’23.4” E), Bahawalpur (29°41’72.8"N, 71°66’86.4” E), and Muzaffargarh (30°10’03.7” N 70°56’44.1” E). Preferably, soil samples were collected from moist sites to assure the existence of active microbial communities. Collected soil samples were then immediately packed in sterilized and clean plastic bags and transferred to the research laboratory, Institute of Soil and Environmental Sciences (ISES), University of Agriculture Faisalabad (UAF), Pakistan, and were kept at 4 °C before further use. Thereafter, the selected PHCs-contaminated soil samples were used to set up the microcosm experiments to enrich PHCs degrading microbial culture.

## Microcosm enrichment and subculture experiments

The selected PHCs-contaminated soil samples were used to set up the first enrichment microcosm experiment. Aerobic incubation was used to enrich microbial consortia in 200-mL Erlenmeyer flasks with 90 mL of C-free mineral salt medium (MSM) having different chemical compositions as described by Suja et al. [34]. After thorough mixing, the final pH of MSM was determined (i.e., 7.0 ± 0.2). Finally, MSM along with other utilities were autoclaved for the setup of microcosm experiments.

In the first enrichment experiment, diesel (1% v/v) was added as a source of PHCs, and acetone was used as a carrier solvent. Subsequently, each 200-mL Erlenmeyer flask, containing 90 mL of autoclaved MSM, was supplied with 10.0 ± 0.5 g of soil (as inoculum) that had been collected from the contaminated spots. Autoclaved controls were also made to evaluate the existence of any abiotic degradation of PHCs. Lastly, flasks were kept at room temperature (25.0 ± 0.5 °C) in the dark on a rotary shaker at 150 rpm [35]. For the first enrichment experiments, at least triplicate microcosms were prepared for the soil of each contaminated site.

After a successful first enrichment experiment (by checking the remaining PHCs contents and subsequent microbial growth), sequential subculturing was done up to four times to enrich PHCs degrading microbial culture. For each subculturing, 10 mL culture (from the previous enrichment) was added to 90 mL freshly prepared (autoclaved) MSM, and diesel (1% v/v) was also added to the medium. All other incubation conditions were kept the same as described above. Microbial consortia were enriched after sequential transfers and/or subcultures.

## Determination of microbial growth and remaining petroleum hydrocarbon contents

A spectrophotometer (Thermo Electron Corporation, Evolution-300LC, England) was used to estimate the growth of microbes by determining the optical density (OD) at 600 nm following the methods described by [35]. For the first enrichment, microbial growth was not measured due to the presence of soil particles (which could significantly affect OD_600_ values). Similarly, a portable hydrocarbons analyzer (PHA-100 plus, PETROSENSE, CA, USA) was used to determine the remaining contents of PHCs in all microcosm experiments by following the detailed methodology described in our previous studies [36, 37].

## Culture optimization experiments

For the culture optimization experiment, the two best-enriched cultures (from site 1 and site 5) were further selected based on higher microbial growth (in terms of OD_600_ value) and more PHCs degradation (in percentage) basis as compared to site 2, site 3 and site 4. To optimize the performance of PHCs degrading indigenous microbial consortia (developed through sequential transfers and sub-culturing), a separate set of experiments was conducted. For this purpose, PHCs degradation and microbial growth of developed consortia were assessed with different levels of pH and temperatures.

Briefly, 10 mL of culture (at 0.5 OD_600_) from enriched culture was added in the same growth medium (C-free MSM) as described above. For the pH experiment, different pH levels (i.e., 3.5, 5.5, 7.0, 8.5, and 9.5) were obtained by adding an appropriate amount of an acid (HCl, 0.01 N) or an alkali (NaOH, 0.01 N). In the case of the temperature experiment, microcosm bottles were kept at different temperatures (i.e., 4, 15, 25, and 45 °C). All microcosm flasks (except temperature optimization experiment) were kept on a rotating shaker with continuous shaking (@150 rpm) in the dark at 25.0 ± 0.5 °C [35].

## Isolation of PHCs-degrading bacterial strains from enriched culture by agar plate method

After successfully completing the initial trials, the best two sites (i.e., site 1 and site 5) were further used in isolation technique via the agar plate method. Briefly, C-free MSM was prepared once again, and agar was added to it. This media was poured into petri plates after being autoclaved and then 100 µL diesel (which is filter sterilized) was added to all plates and spread equally with the help of an autoclaved spreader. Thereafter, 1 µL microbial culture was properly placed all over the plates to ensure homogeneity. These petri plates were then incubated at room temperature of 25 ± 3 ^○^C for 72 h and afterwards, the plates were carefully examined to see the colony formations. The bacterial strains were isolated from the plates by observing their colony size and growth patterns. The same procedure was repeated to cultivate the pure bacterial strains on separately prepared MSM-agar plates.

### Characterization and identification of selected PHCs degrading bacterial isolates

A total of 25 bacterial isolates (9 from site 1 and 16 from site 5) were selected initially based on their morphological characteristics and colony appearance. These bacterial isolates were then used in various biochemical assays to check their potential as a plant growth promoter and stress-tolerant nature. Thereafter, a total of 9 bacterial isolates (four from site 1 and five from site 5) were further selected based on subjected biochemical assays such as catalase, oxidase, siderophore, ACC-deaminase, phosphatase, and exopolysaccharides. Details of the mentioned attributes were already presented in our previous work [36]. After characterization of bacterial isolates based on plant growth promotion, a sufficient amount of each bacterial isolate was collected in microtubes (LB media with 30% glycerin solution) and stored at -80 °C for preservation. For identification of isolated strains, the nine best isolates were then sent to Macrogen, Inc, South Korea for Sanger sequencing, targeting the 16 S rRNA gene (Fig. S1).

## Preparation of bacterial consortia

The four bacterial strains including *Alcaligenes faecalis* strain MH-2 (ON714529), *Alcaligenes* sp. strain MH-3 (ON714530), *Achromobacter denitrificans* strain MH-6 (ON714531), *Sphingobacterium spiritivorum* strain MH-9 (ON714532) were mixed together (by adding 4.0 mL of each strain from their enriched culture) in fresh autoclaved MSM (980 mL) to form bacterial consortia 1. Similarly, five bacterial strains including *Sphingobacterium spiritivorum* (ON714533 strain MH-10), *Achromobacter xylosoxidans* strain MH-13 (ON714534), *Stenotrophomonas* sp. strain MH-18 (ON714535), *Alcaligenes faecalis* strain MH-22 (ON714536) and *Stenotrophomonas rhizophila* strain MH- 24 (ON714537) were also mixed (by adding 5.0 mL of each strain from their enriched culture) in newly prepared and autoclaved 980 mL MSM to construct bacterial consortium 2 (BC2). These two bacterial consortia (i.e., BC1 and BC2) were tested for PHCs degradation by rhizoremediation experiment with chickpea in PHCs contaminated soils.

## Experimental setup

For the pot experiment, the soil was collected from the agronomic research area of ISES, UAF Faisalabad. Initial physicochemical attributes of soil were examined by following the prescribed methodologies of Estefan et al. [38]. The seeds of the chickpea, variety NIAB-CH-2016, were obtained from the Nuclear Institute for Agriculture and Biology (NIAB), Faisalabad, Pakistan. Thereafter, a pot trial was carried out in the wirehouse of ISES, UAF, Faisalabad, Pakistan to evaluate the effect of bacterial consortia (i.e., BC1 and BC2) on the rhizoremediation of PHCs and plant growth enhancement of chickpea plant (variety, NIAB-CH-2016) in soil artificially spiked with 1.5% and 3.0% diesel. Six, surface sterilized seeds (un-inoculated or inoculated with culture) of chickpea were sown in respective pots containing 10 kg of unsterilized contaminated or uncontaminated soils. The suggested amount of N (20 kg ha^− 1^ as urea), P (60 kg ha^− 1^ as single super-phosphate), and K (60 kg ha^− 1^ as sulfate of potash) for chickpea was added to all pots [36]. The experiment was carried out with fifteen distinct treatments and three replications, including three levels of PHCs (0.0%, 1.5% and 3.0%) in different combinations with two bacterial consortia BC1 and BC2 (i.e., 50 mL). A total of forty-five experimental pots were placed in wire house by complete randomization and the treatment details of the experiment are presented in Table S1.

### Determination of plant growth attributes

Seedling emergence was observed from the 2nd day of sowing, but the emergence count was done on the 3rd day because sprouting was not clear for the count till the 2nd day. The seedling was observed daily until a constant count was achieved. At the time of harvest, the root and shoot lengths were taken by a measuring stick. For plant dry weight, samples were placed in an oven (at 65 °C) till a constant weight was obtained on a portable weight balance. Other growth attributes such as the number of leaves, branches, pods and nodules were counted manually at the time of harvesting.

### Determination of plant physiological attributes

Mature fresh leaves were collected from each plant to determine chlorophyll contents such as chlorophyll a (Chl a), chlorophyll b (Chl b), total chlorophyll (T Chl), and carotenoid by following the standard procedures of Arnon [39]. In brief, 0.5 g of fresh leaves were ground in 5 mL (80%) methanol and the material was centrifugated at 5000 rpm at 20 °C for 10 min. After that, the aliquot was separated and collected in vials to run through a UV-visible spectrophotometer, and the OD was measured at distinct wavelengths such as 480, 645, and 663 nm. The SPAD value of the plants were also taken by using a SPAD meter (SPAD-502 Konica, Minolta Optics, Inc, Japan) after 45 d of seed sowing [40]. Similarly, the photochemical quantum yield (YII), fluorescence yield (Ft), photosynthetically active radiation (PAR) and electron transport rate (ETR) of chickpeas were measured by a photosynthetic yield analyzer in sunny bright day at around 12:00 to 01:00 p.m [8].

To calculate the membrane stability index (MSI), the procedure of Sairam et al. [41] was adopted in which the leachate (ions) from plant leaves was collected in deionized water. In brief, test tubes were filled with 0.2 g of fresh leaf samples and 10 mL of deionized water in 2 sets. The first experimental set having same composition, was kept in a water bath for 30 min at 40 °C, and then EC meter was used to record EC1. While for obtaining EC2, the second experimental set was kept at 100 °C in a water bath for 15 min. The Eq. (1) used for the calculation of MSI is as follows:

1 $$\text{MSI}{\text{ }} = {\text{ }}\left[ {1 - \left( {\text{EC}1/\text{EC}2} \right)} \right]{\text{ }}*{\text{ }}100$$

$$\text{EC}1{\text{ }} = {\text{ }}\text{EC}{\text{ }}\text{obtained}{\text{ }}\text{ from}{\text{ }}\text{the}{\text{ }}\text{first}{\text{ }}\text{set}{\text{ }}\text{of}{\text{ }}\text{samples}$$

$$\text{EC}1{\text{ }} = {\text{ }}\text{EC}{\text{ }}\text{obtained}{\text{ }}\text{ from}{\text{ }}\text{the}{\text{ }}\text{second}{\text{ }}\text{set}{\text{ }}\text{of}{\text{ }}\text{samples}$$

Similarly, the relative water contents (RWC) were also calculated by following the methods outlined by Sairam et al. [41]. Precisely, fresh leaves (FW) having 0.5 g weight were dipped immediately in distilled water to gain maximum turgidity for approximately 4 h. Thereafter, these samples were taken out and the surface of the leaf samples was dried using tissue paper immediately, then weight the samples to gain fully turgid weight (TW). Afterwards, these samples were oven-dried for 2 d at 65 °C to obtain the dry weight (DW). The equation used for the calculation of RWC is as follows:

2 $$[{\text{RWC = [(FW - DW) / (TW - DW)]}}$$

#### Determination of antioxidative attributes of chickpea plants

To measure the total proline contents of plant leaves, the standard protocols of Bates et al. [42] were followed and the brief methodology has already been provided in our previous study [37]. For catalase (CAT) activity, the methodologies described by Ahmad et al. [43] were adopted. For this purpose, an exact amount of 10.4 mL mixture which contains enzyme extract (200 µL), 0.3 M H_2_O_2_ (200 µL) and EDTA (0.002 M) with 0.05 M phosphate buffer (10 mL). The pH of the mixture was 7.0. A spectrophotometer at 240 nm was used to measure the induced reduction because of the disappearance of H_2_O_2_. Similarly, for superoxide dismutase (SOD) activity, the reduction in absorbance of nitroblue tetrazolium chloride was observed at 560 nm [44]. Peroxidase (POD) activity of leaves was measured by following an established protocol of Angelini et al. [45] to monitor the absorbance at 436 nm to determine the conversion of guaiacol to tetra-guaiacol.

### Determination of nutrients attributes of chickpea plants and residual PHCs in soil

Plants nutrient analysis were done by following the methodologies of Estefan et al. [38] in which the total K, P, and N contents of digested plant samples were determined via flame photometer, spectrophotometer and Kjeldahl apparatus respectively. In the same way, the residual contents of PHCs in soil were estimated by a portable hydrocarbon analyzer (PHA-100 plus, PETROSENSE, CA, USA) following the detailed protocols and procedures described in our previous works [36, 37].

### Statistical analysis

The F-test was performed to the obtained data to check the level of significance among different treatment means, and the honest significant difference (HSD) test was applied to check the significance among various treatment means by using Statistix 8.1 software [46] The analysis of variance (ANOVA) was applied to the acquired data and the variations in mean values (*n* = 3) were presented by the standard error. Correlation matrix and principal component analyses (PCA) were also done to check the correlation between different treatments by employing Origin 2022b software [47].

## Results

### Physicochemical attributes of soil

The texture of the soil was sandy loam with 53% sand, 25% silt and 22% clay contents. The pH, electrical conductivity (EC), exchangeable cations, and organic matter of soil were 7.95 ± 0.17, 1.07 ± 0.28 dS m^**–**1^, 16.3 ± 1.43 mmolc L^**–**1^, and 0.73 ± 0.05%, respectively. Moreover, total N, available P, and K were 180 ± 3.20 mg kg^**–**1^, 86.7 ± 1.09 mg kg^**–**1^, and 58.4 ± 2.35 mg kg^**–**1^, respectively. However, the PHCs contents in soil were not detectable.

### Microcosm enrichment and culture optimization experiments

**Fig. 1:**
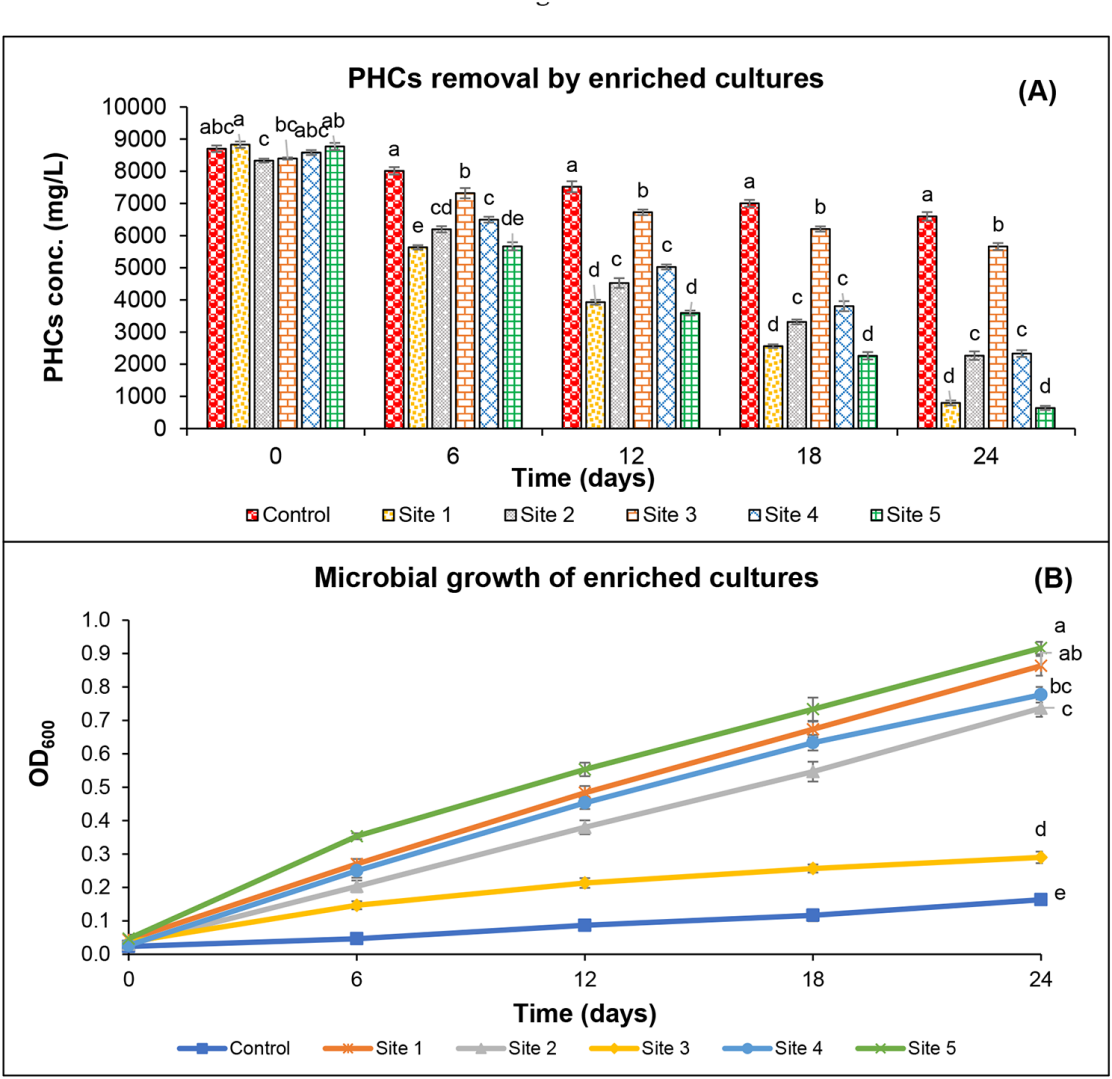
PHCs removal (**A**) and growth (**B**) by microbial cultures enriched (after three sequential transfers) from soils collected from five different contaminated sites. Each column represents the mean value of treatment, and each bar represents the standard error within the same treatment. The small alphabetical letters present the statistical variation among different means at the same time point. PHCs, petroleum hydrocarbons; Site 1, soil collected from PHCs contaminated site in Abbottabad; Site 2, soil collected from PHCs contaminated site in Rawalpindi, Site 3; soil collected from PHCs contaminated site in Faisalabad; Site 4, soil collected from PHCs contaminated site in Bahawalpur; Site 5, Soil collected from PHCs contaminated site in Muzaffargarh

In the first enrichment experiment, after two weeks of incubation, the removal of PHCs was significantly increased in all microcosm experiments (except site 3) than that of autoclaved control (Fig. S2 A). In the subsequent subculturing, PHCs remediation and microbial growth on PHCs in MSM were further improved [Fig. S2 (B and C)]. Moreover, after three sequential transfers, the soil-free enriched culture was capable to remediate 92.0% (site 1), 66.3% (site 2), 43.4% (site 3), 61.9% (site 4), and 93.5% (site 5) of initially-added concentration of PHCs after 24 d incubation (Fig. 1A). Here, the microbial growth from site 1 and site 5 showed higher growth in terms of OD_600_ (Fig. 1B and Fig. S3). The culture optimization experiments showed that enriched cultures were capable to remediate PHCs at a wide range of pH and temperature (Figs. S4 and S5); however, cultures demonstrated highest removal at neutral pH and room temperature (i.e., 25 °C) as presented in Figs. S4 and S5. In general, during culture optimization experiments, enriched cultures obtained from site 1 and site 5 performed better in case of growth (in terms of OD_600_) and PHCs removal at all tested levels of pH and temperature (Figs. S4 and S5) and therefore further selected for screening, plant growth promotion and rhizoremediation experiments.

### Plant growth promotion and rhizoremediation experiments

After the culture optimization experiment, four bacterial strains were screened and selected from site 1 and five from site 5 and mixed to construct BC1 (from site 1) and BC2 (from site 5). Thereafter, the prepared bacterial consortia (BC1 and BC2) were used with chickpea plants in soil artificially spiked with diesel. The results of the plant growth promotion and rhizoremediation experiment are provided in detail below.

### Seedling emergence and plant growth attributes

The PHCs contamination substantially reduced seedling emergence **(**Table 1**)**. Furthermore, regardless of the treatments, most of the seedling were emerged after 2 days of seed sowing. However, the addition of BC significantly improved the seedling emergence than that of control (without PHCs and BC). In uncontaminated soil, the addition of BC1 (5.89%) and BC2 (11.1%) resulted in more sprouting rather than uninoculated control **(**Table 1**)**. In the case of 1.5% PHCs contamination, the addition of BC1 and BC2 caused an increase of 10.6% and 16.7%, respectively in seedling emergence showing the critical role that microbes play in germination. However, at higher level of contamination (i.e. 3.0% PHCs) both bacterial consortia did not show any significant improvement.

**Table 1:** Effect of bacterial consortia addition on seedling emergence of chickpea under petroleum hydrocarbon stress

Treatments	Seedling emergence (%)		
	3 DAS	4 DAS	5 DAS
Control	72.2 ± 5.54a	72.2 ± 5.54ab	88.9 ± 5.57ab
PHCs 1.5%	44.4 ± 5.57ab	50.0 ± 9.65bc	55.6 ± 5.57bc
PHCs 3.0%	27.8 ± 5.54b	38.9 ± 5.57c	50.0 ± 9.65c
PHCs 0.0%+BC1	72.2 ± 5.54a	88.9 ± 5.57a	94.4 ± 5.57a
PHCs 1.5%+BC1	50.0 ± 9.65ab	55.5 ± 5.57bc	66.7 ± 9.62abc
PHCs 3.0%+BC1	33.3 ± 9.62b	44.4 ± 5.57bc	55.5 ± 5.57bc
PHCs 0.0%+BC2	72.2 ± 5.54a	88.9 ± 5.57a	100 ± 0.00a
PHCs 1.5%+BC2	50.0 ± 9.65ab	61.1 ± 5.57abc	66.7 ± 9.62abc
PHCs 3.0%+BC2	38.8 ± 5.57ab	44.4 ± 5.57bc	55.6 ± 5.57bc

Values are the means of three replicates ± standard errors. Means with different letters in a column are significantly different at *P* ≤ 0.05 according to HSD test. BC1, bacterial consortium 1 of selected bacterial strains *A. faecalis*, *Alcaligenes* sp., *A*. *denitrificans* and *S*. *spiritivorum*; BC2, bacterial consortium 2 of selected bacterial strains *S*. *spiritivorum*, *A*. *xylosoxidans*, *Stenotrophomonas* sp., *A. faecalis* and *S*. *rhizophila*; PHCs, petroleum hydrocarbons; DAS, days after sowing

**Fig. 2:**
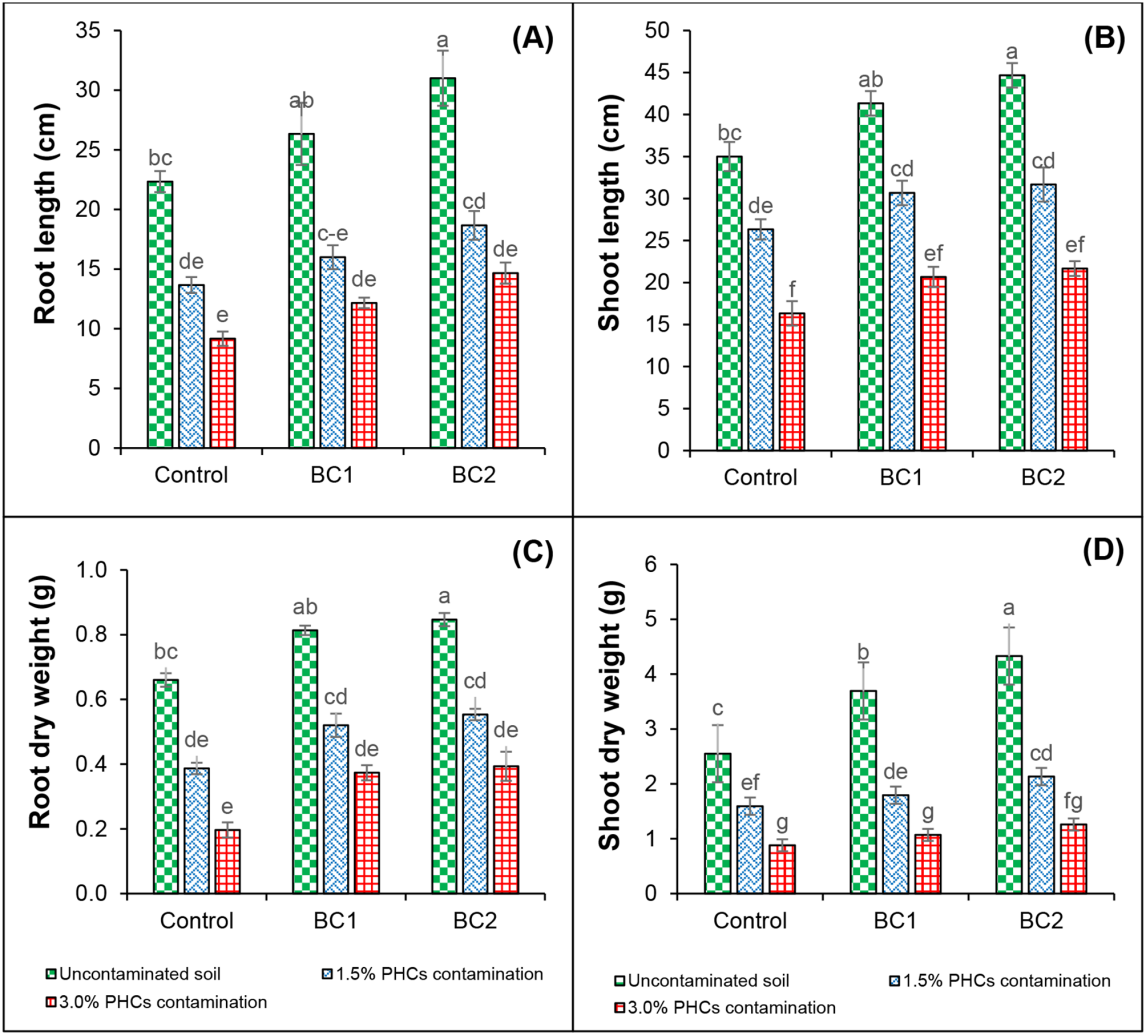
Effect of bacterial consortia of selected bacterial strains on the root length (**A**) shoot length (**B**), root dry weight (**C**) and shoot dry weights (**D**) of chickpea grown under hydrocarbons contamination. Standard errors are represented by bars, and columns represent the means of triplicate values. PHCs, petroleum hydrocarbons; BC1, bacterial consortium 1 of selected bacterial strains *A. faecalis*, *Alcaligenes* sp., *A*. *denitrificans* and *S*. *spiritivorum*; BC2, bacterial consortium 2 of selected bacterial strains *S*. *spiritivorum*, *A*. *xylosoxidans*, *Stenotrophomonas* sp., *A. faecalis* and *S*. *rhizophila*,

The PHCs showed significant phytotoxicity (*P* ≤ 0.05) to chickpea plants and reduced the growth attributes (Fig. 2and Table 2). Under PHCs contamination (1.5% and 3.0%), a significant decrease was noticed in root length (38.8% and 58.9%), shoot length (24.8% and 53.3%), root dry weight (34.7% and 53.4%), shoot dry weight (37.5% and 65.5%), number of nodules (50.0% and 82.1%), number of pods (41.2% and 88.1%), number of stems (50.0% and 60.0%), and number of compound leaves per plants (44.3% and 73.7%), when compared with uncontaminated and uninoculated control. The treatments containing microbial cultures in the absence of PHCs stress improved all these mentioned growth attributes as compared to the control (uncontaminated and uninoculated control).

**Table 2:** Effect of bacterial consortia on morphological attributes of chickpea plants grown under petroleum hydrocarbon stress

Treatments	NN	NP	NS	NCL
Control	9.33 ± 0.88^b^	22.7 ± 0.88^b^	3.33 ± 0.33^abc^	73.0 ± 4.93^bc^
PHCs 1.5%	4.67 ± 0.67^cde^	7.67 ± 0.88^d^	1.67 ± 0.33^cd^	42.3 ± 2.41^def^
PHCs 3.0%	1.67 ± 0.88^e^	2.67 ± 0.33^d^	1.33 ± 0.33^d^	20.0 ± 2.08^f^
PHCs 0.0%+BC1	14.3 ± 1.20^a^	26.7 ± 0.88^ab^	4.33 ± 0.33^ab^	90.3 ± 6.37^ab^
PHCs 1.5%+BC1	6.67 ± 0.33^bcd^	14.7 ± 0.88^c^	2.33 ± 0.33^cd^	55.0 ± 4.17^cde^
PHCs 3.0%+BC1	2.67 ± 0.33^de^	3.67 ± 0.33^d^	1.67 ± 0.33^cd^	26.7 ± 2.34^f^
PHCs 0.0%+BC2	16.7 ± 0.88^a^	30.7 ± 2.61^a^	4.67 ± 0.33^a^	107 ± 10.8^a^
PHCs 1.5%+BC2	7.33 ± 0.88^bc^	16.3 ± 0.67^c^	2.67 ± 0.33^bcd^	62.3 ± 5.21^bcd^
PHCs 3.0%+BC2	3.67 ± 0.88^cde^	3.67 ± 0.33^d^	1.67 ± 0.33^cd^	32.7 ± 2.03^ef^

Values are the means of three replicates ± standard errors. Means with different letters in a same column are significantly different at *P* ≤ 0.05 according to HSD test. NN, number of nodules; NP, number of pods per plant; NS, number of stems per plant; NCL, number of compound leaves per plant; BC1, bacterial consortium 1 of selected bacterial strains *A. faecalis*, *Alcaligenes* sp., *A*. *denitrificans* and *S*. *spiritivorum*; BC2, bacterial consortium 2 of selected bacterial strains *S*. *spiritivorum*, *A*. *xylosoxidans*, *Stenotrophomonas* sp., *A. faecalis* and *S*. *rhizophila*; PHCs, petroleum hydrocarbons

In the absence of PHCs contamination and the inclusion of bacterial consortia (BC1 and BC2), the root length (15.2% and 27.9%), shoot length (15.3% and 21.6%), root dry weight (17.5% and 24.0%), shoot dry weight (30.9% and 41.9%), number of nodules (34.9% and 44.0%), number of pods (15.0% and 26.1%), number of stems (23.1% and 28.6%) and number of compound leaves per plants (15.9% and 28.8%) than that of uncontaminated and uninoculated control (Fig. 2and Table 2). Similarly, at 1.5% PHCs contamination, the inclusion of both cultures (BC1 and BC2) reduced the phytotoxicity of PHCs on plant growth attributes and improved the root length (14.5% and 26.7%), shoot length (14.1% and 16.8%), root dry weight (19.2% and 20.3%), shoot dry weight (11.2% and 25.3%), number of nodules (30.0% and 36.4%), number of pods (9.09% and 18.4%), number of stems (28.6% and 37.5%) and number of leaves (compound) per plant (23.1% and 32.1%), respectively in comparison with respective uninoculated control. Likewise, at 3.0% PHCs contamination, the addition of both cultures (BC1 and BC2) increased the root length (24.6% and 37.5%), shoot length (20.9% and 24.6%), root dry weight (19.6% and 23.7%), shoot dry weight (17.8% and 30.1%), number of nodules (37.5% and 54.5%), number of pods (27.3% and 27.3%), number of stems (20.0% and 20.0%), and number of leaves (compound) per plant (25.0% and 38.8%), respectively over their respective uninoculated control (Fig. 2and Table 2).

#### Physiological attributes of chickpea plants

**Fig. 3:**
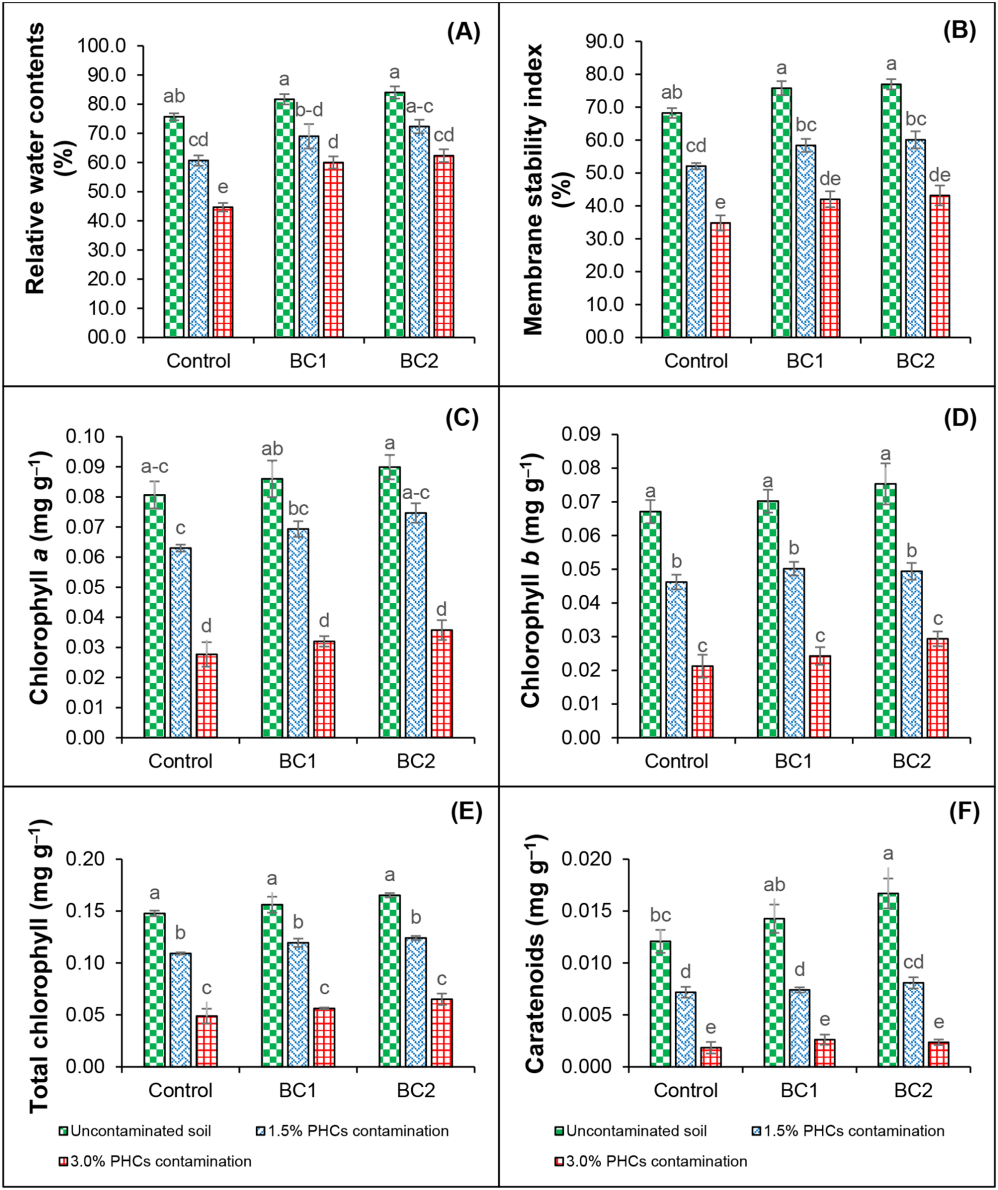
Effect of bacterial consortia on chlorophyll *a* (**A**), chlorophyll *b* (**B**), total chlorophylls (**C**), carotenoids (**D**), relative water contents (**E**) and membrane stability index (**F**) of fresh leaves of chickpea grown under hydrocarbons stress. Standard errors are represented by bars, and columns represent the means of triplicate values. PHCs, petroleum hydrocarbons; BC1, bacterial consortium 1 of selected bacterial strains *A. faecalis*, *Alcaligenes* sp., *A*. *denitrificans* and *S*. *spiritivorum*; BC2, bacterial consortium 2 of selected bacterial strains *S*. *spiritivorum*, *A*. *xylosoxidans*, *Stenotrophomonas* sp., *A. faecalis* and *S*. *rhizophila*

Petroleum hydrocarbon phytotoxicity significantly (*P* ≤ 0.05) reduced the RWC and MSI of chickpea plants. In the case of 1.5% PHCs contamination, the RWC and MSI were reduced by 19.8% and 23.7%, respectively compared to uninoculated and uncontaminated control. Likewise, at 3.0% PHCs contamination, a decrease of 40.9% (in MSI) and 48.9% (RWC) has been observed when compared with unamended control (without PHCs and BC). However, the inclusion of bacterial consortia (BC1 and BC2) produced (7.34% and 9.92%), (9.97% and 11.3%) higher RWC and MSI as compared to control treatment. Similarly, an increase of 12.1% and 16.1% in MSI and 10.8% and 13.3% in RWC was also observed in BC1 and BC2 applied treatments, respectively under 1.5% PHCs contamination, over their respective uninoculated control (with 1.5% PHCs). Additionally, in the case of 3.0% PHCs contamination, there was about 25.6% and 17.1% increase in BC1 applied treatment (in case of MSI) and 28.3% and 19.4% increase in BC2 applied treatment (in case of RWC) was noticed than that of their uninoculated control (with 3.0% PHCs) (Fig. 3).

In the present study, the results revealed that the PHCs significantly (*P* ≤ 0.05) reduced the Chl a, Chl b, T Chl, and carotenoid contents of chickpea plants (Fig. 3). The Chl a, Chl b, T Chl and carotenoid contents were reduced by 21.9% and 65.7%, 31.1% and 68.4%, 26.1% and 66.9%, 40.5% and 84.8% under 1.5% and 3.0% PHCs contamination respectively, in comparison to uncontaminated control (without PHCs and BC). The treatments containing bacterial consortia (BC1 and BC2) generated higher Chl a (6.20% and 10.3%), Chl b (4.43% and 10.9%), T Chl (5.41% and 10.6%) and carotenoids contents (15.3% and 27.6%) than that of uninoculated and uncontaminated control. However, the treatments containing 1.5% PHCs contamination with the application of bacterial consortia i.e., BC1 and BC2, increased the Chl a, Chl b, T Chl and carotenoid contents by 9.13% and 15.6%, 7.91% and 6.46%, 8.62% and 11.9% and 3.01% and 11.2%, respectively over their uninoculated control (with 1.5% PHCs but without BC) (see Fig. 3). Additionally, the application of BC1 and BC2 in 3.0% PHCs contamination, showed an increase of 13.5% and 22.6% in Chl a, 12.5% and 27.6% in Chl b, 13.1% and 24.9% in T Chl, and 29.6% and 22.1% in carotenoid contents, respectively as compared to their uninoculated control (with 3.0% PHCs but without BC).

The toxic effects of PHCs on chickpea plants significantly (*P* ≤ 0.05) reduced the SPAD value, Ft, PAR, YII, and ETR of chickpea plants (Table 3). In the case of PHCs contamination (1.5% and 3.0%), a significant decrease was observed in SPAD value (29.7% and 44.4%), Ft (29.7% and 55.5%), PAR (8.19% and 17.9%), YII (13.1% and 37.3%), ETR (17.2% and 29.4%), over their unamended control (in the absence of PHCs and BC). The treatments containing both BC produced higher physiological yield attributes as compared to uninoculated and uncontaminated control (without BC and PHCs). The SPAD value (5.29% and 10.5%), Ft (21.1% and 35.5%), PAR (4.92% and 6.83%), YII (4.59% and 17.2%) and ETR (23.6% and 39.2%) of chickpea plants was also improved in the presence of bacterial consortia (BC1 and BC2) (see Table 3). Similarly, the inclusion of BC1 and BC2 in 1.5% PHCs contamination resulted in an increase of 22.1% and 25.9% in SPAD value, 26.1% and 29.3% in Ft, 2.76% and 4.13% in PAR, 2.86% and 12.9% in YII and 13.5% and 24.4% in ETR over their uninoculated control (with 1.5% PHCs but without BC). Likewise, BC1 and BC2 applied treatments showed an improved SPAD value (by 15.1% and 20.5%), Ft (by 26.6% and 37.4%), PAR (by 7.74% and 10.2%), YII (by 13.8% and 18.6%) and ETR (by 12.6% and 26.5%), as compared to their relative control (with 3.0% PHCs but without BC).

**Table 3:** Effect of bacterial consortia on physiological attributes of chickpea plants grown under petroleum hydrocarbon stress

Treatments	SPAD value	Ft (µmolm^–2^s^–1^)	PAR (µmolm^–2^s^–1^)	YII (µmolm^–2^s^–1^)	ETR (µmolm^–2^s^–1^)
Control	41.7 ± 1.18^ab^	253 ± 32.1^bc^	818 ± 9.71^ab^	0.74 ± 0.04^b^	46.8 ± 6.06^bcd^
PHCs 1.5%	29.3 ± 1.66^cd^	178 ± 18.0^cde^	751 ± 15.4^c^	0.64 ± 0.02^bc^	38.7 ± 2.26^cd^
PHCs 3.0%	23.2 ± 1.79^d^	112 ± 13.4^e^	671 ± 13.3^d^	0.46 ± 0.02^d^	33.0 ± 2.30^d^
PHCs 0.0%+BC1	44.1 ± 1.99^ab^	320 ± 22.9^ab^	860 ± 7.10^a^	0.77 ± 0.03^ab^	61.3 ± 4.87^ab^
PHCs 1.5%+BC1	37.7 ± 1.59^bc^	240 ± 16.2^bcd^	772 ± 8.33^bc^	0.66 ± 0.4^bc^	44.8 ± 2.14^bcd^
PHCs 3.0%+BC1	27.3 ± 2.15^d^	153 ± 6.09^de^	728 ± 29.3^cd^	0.54 ± 0.02^cd^	37.8 ± 2.14^cd^
PHCs 0.0%+BC2	46.7 ± 1.22^a^	392 ± 15.9^a^	878 ± 9.53^a^	0.89 ± 0.03^a^	77.0 ± 2.61^a^
PHCs 1.5%+BC2	39.6 ± 1.39^ab^	251 ± 7.63^bc^	783 ± 12.2^bc^	0.74 ± 0.01^b^	51.3 ± 2.43^bc^
PHCs 3.0%+BC2	29.2 ± 2.63^cd^	179 ± 11.1^cde^	747 ± 16.8^c^	0.57 ± 0.01^cd^	44.9 ± 3.49^bcd^

Values are the means of three replicates ± standard errors. Means with different letters in a column are significantly different at *P* ≤ 0.05 according to HSD test. SPAD, Soil plant analysis and development value; Ft, fluorescence yield; PAR, photosynthetically active radiation; YII, photochemical quantum yield; ETR, electron transport rate; BC1, bacterial consortium 1 of selected bacterial strains *A. faecalis*, *Alcaligenes* sp., *A*. *denitrificans* and *S*. *spiritivorum*; BC2, bacterial consortium 2 of selected bacterial strains *S*. *spiritivorum*, *A*. *xylosoxidans*, *Stenotrophomonas* sp., *A. faecalis* and *S*. *rhizophila*; PHCs, petroleum hydrocarbons

### Antioxidative enzymes of chickpea plants

The phytotoxicity of PHCs to chickpea plants significantly (*P* ≤ 0.05) increased the proline, CAT, SOD, and POD contents (Table 4). The proline, CAT, SOD, and POD contents were increased by 46.4% and 62.2%, 61.6% and 74.3%, 32.5% and 45.6%, 41.4% and 51.9% under 1.5% and 3.0% PHCs contamination respectively, over their uncontaminated control (without PHCs and BC). However, no significant improvement in proline contents was observed in uncontaminated and inoculated treatments (treatments without PHCs but with BC). However, the treatments containing 1.5% PHCs contamination with the presence of BC1 and BC2, a substantial decrease of 16.9% and 23.6% in proline, 21.9% and 24.0% CAT, 18.9% and 23.7% in SOD and 17.8% and 22.7% in POD contents, respectively was observed in comparison to uninoculated control (with 1.5% PHCs but without BC) (Table 4). Similarly, the application of bacterial consortia i.e., BC1 and BC2 in 3.0% PHCs contamination, showed a reduction of 13.5% and 22.6% in proline, 12.5% and 27.6% in CAT, 13.1% and 24.9% SOD, and 29.6% and 22.1% POD contents, respectively were examined as compared to respective control (with 3.0% PHCs but without BC).

**Table 4:** Effect of bacterial consortia in producing various antioxidants in chickpea plants grown under petroleum hydrocarbons stress

Treatments	PC (µmole g^–1^ FW)	CAT (U mg^–1^ protein FW)	SOD (U mg^–1^ protein FW)	POD (U mg^–1^ protein FW)
Control	18.9 *±* 1.51^d^	3.49 *±* 0.24^d^	11.1 *±* 0.91^cd^	65.9 *±* 3.64^d^
PHCs 1.5%	35.4 *±* 2.05^bc^	9.08 *±* 0.75^bc^	16.4 *±* 0.49^b^	112 *±* 4.95^b^
PHCs 3.0%	50.1 *±* 1.95^a^	13.6 *±* 1.00^a^	20.3 *±* 1.24^a^	137 *±* 2.50^a^
PHCs 0.0%+BC1	19.6 *±* 0.83^d^	3.28 *±* 0.09^d^	11.1 *±* 0.86^cd^	64.2 *±* 3.04^d^
PHCs 1.5%+BC1	29.4 *±* 1.79^cd^	7.09 *±* 0.29^c^	12.9 *±* 0.49^c^	92.4 *±* 3.43^c^
PHCs 3.0%+BC1	43.6 *±* 3.34^ab^	11.1 *±* 0.76^ab^	16.5 *±* 0.54^b^	122 *±* 2.14^ab^
PHCs 0.0%+BC2	19.7 *±* 1.94^d^	3.26 *±* 0.08^d^	8.73 *±* 0.82^d^	67.4 *±* 2.54^d^
PHCs 1.5%+BC2	27.1 *±* 1.73^cd^	6.90 *±* 0.24^c^	11.6 *±* 0.48^c^	86.9 *±* 3.42^c^
PHCs 3.0%+BC2	41.9 *±* 3.03^ab^	9.45 *±* 0.54^bc^	15.5 *±* 0.47^b^	118 *±* 3.63^b^

Values are the means of three replicates ± standard errors. Means with different letters are significantly different at *P* ≤ 0.05 according to HSD test. PC, proline contents; CAT, catalase; SOD, superoxide dismutase; POD, peroxidase; BC1, bacterial consortium 1 of selected bacterial strains *A. faecalis*, *Alcaligenes* sp., *A*. *denitrificans* and *S*. *spiritivorum*; BC2, bacterial consortium 2 of selected bacterial strains *S*. *spiritivorum*, *A*. *xylosoxidans*, *Stenotrophomonas* sp., *A. faecalis* and *S*. *rhizophila*; PHCs, petroleum hydrocarbons; FW, fresh weight

#### Nutrient acquisition in plants and removal of hydrocarbons from soil

The toxic impacts of PHCs on chickpea plants was significantly (*P* ≤ 0.05) decrease the N, P, and K contents of chickpea plants (Table 5). The N, P, and K contents were decreased by 36.7% and 56.0%, 31.3% and 49.6% and 26.3% and 42.6%, under 1.5% and 3.0% PHCs contamination, respectively than that of uncontaminated control (without PHCs and BC). However, the treatments having bacterial consortia (i.e., BC1 and BC2) in the absence of PHCs, produced higher N (i.e., 12.6% and 18.3%), P (i.e., 15.1% and 17.8%) and K contents (i.e., 18.3% and 20.5%) than that of uninoculated and uncontaminated control (without BC and PHCs). However, in the case of 1.5% PHCs contamination and with the addition of BC1 and BC2, a decrease of 20.1% and 28.8% in N, 14.5% and 25.9% in P, and 22.8% and 24.4% in K contents, respectively was noticed than respective control (with 1.5% PHCs but without BC) (see Table 5). Additionally, the application of bacterial consortia i.e., BC1 and BC2 in 3.0% PHCs contamination, showed 21.0% and 25.4% higher N, 12.3% and 16.9% higher P, and 18.2% and 27.7% higher K contents, respectively as compared to their respective control (with 3.0% PHCs but without BC).

**Table 5:** Effect of bacterial consortia on the uptake of macronutrients in chickpea plants grown under petroleum hydrocarbon stress

Treatments	Nutrient uptake (mg kg^–1^) by plants		
	Nitrogen	Phosphorus	Potassium
Control	209 *±* 6.13^bc^	110 *±* 12.0^ab^	147 *±* 11.6^ab^
PHCs 1.5%	132 *±* 7.8^ef^	75.5 *±* 4.71^bcd^	108 *±* 14.8^bc^
PHCs 3.0%	91.7 *±* 9.84^f^	55.1 *±* 6.53^d^	84.1 *±* 4.37^c^
PHCs 0.0%+BC1	238 *±* 16.1^ab^	129 *±* 5.67^a^	179 *±* 8.69^a^
PHCs 1.5%+BC1	165 *±* 7.69^de^	88.4 *±* 8.33^bcd^	140 *±* 9.93^ab^
PHCs 3.0%+BC1	116 *±* 4.20^f^	62.8 *±* 5.54^d^	103 *±* 6.59^bc^
PHCs 0.0%+BC2	255 *±* 10.9^a^	134 *±* 7.36^a^	184 *±* 6.23^a^
PHCs 1.5%+BC2	185 *±* 8.21^de^	102 *±* 7.39^abc^	143 *±* 11.5^ab^
PHCs 3.0%+BC2	123 *±* 3.34^f^	66.3 *±* 4.97^cd^	116 *±* 6.59^bc^

Values are the means of three replicates ± standard errors. Means with different letters are significantly different at *P* ≤ 0.05 according to HSD test. BC1, bacterial consortium 1 of selected bacterial strains *A. faecalis*, *Alcaligenes* sp., *A*. *denitrificans* and *S*. *spiritivorum*; BC2, bacterial consortium 2 of selected bacterial strains *S*. *spiritivorum*, *A*. *xylosoxidans*, *Stenotrophomonas* sp., *A. faecalis* and *S*. *rhizophila*; PHCs, petroleum hydrocarbons

**Fig. 4:**
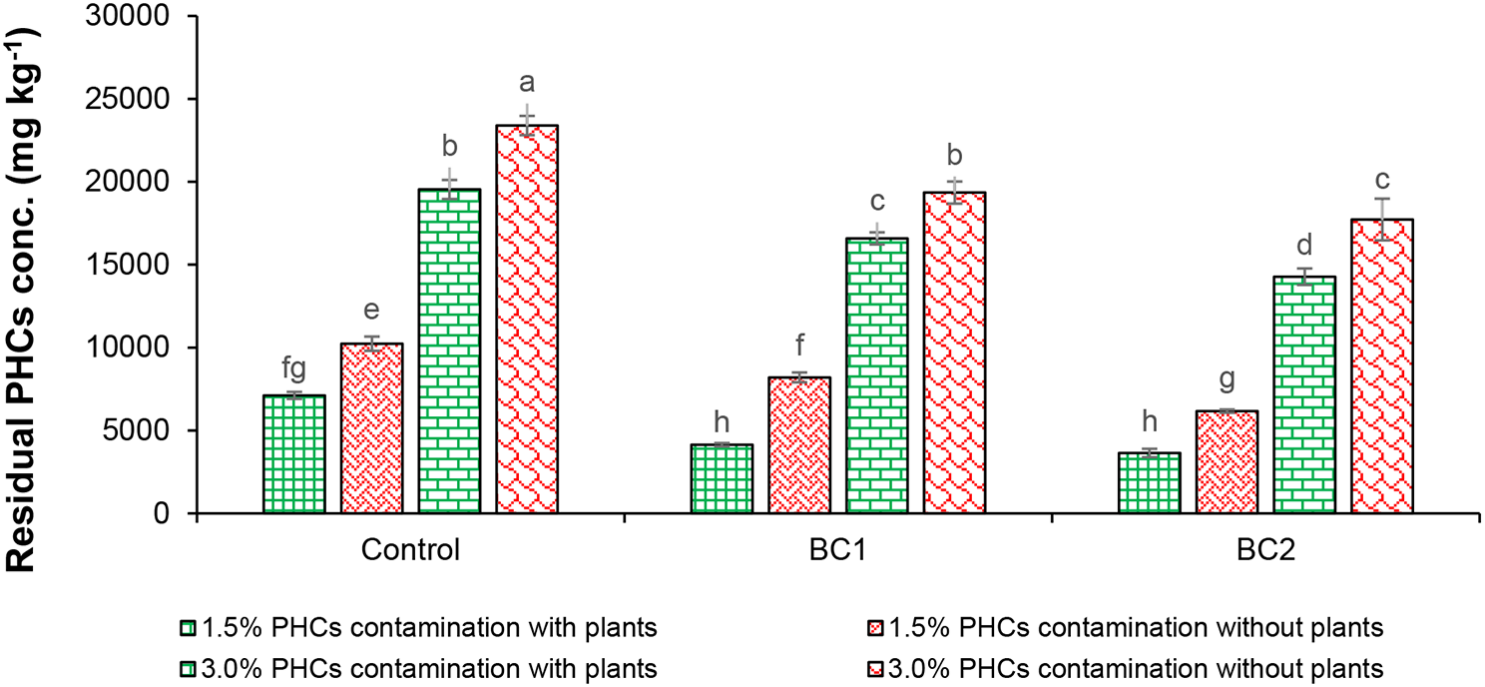
Effect of bacterial consortia on PHCs removal from hydrocarbon-contaminated soil in the presence or absence of chickpea plants. Standard errors are represented by bars, and columns represent the means of triplicate values. PHCs, petroleum hydrocarbons; BC1, bacterial consortium 1 of selected bacterial strains *A. faecalis*, *Alcaligenes* sp., *A*. *denitrificans* and *S*. *spiritivorum*; BC2, bacterial consortium 2 of selected bacterial strains *S*. *spiritivorum*, *A*. *xylosoxidans*, *Stenotrophomonas* sp., *A. faecalis* and *S*. *rhizophila*

Overall, in the case of hydrocarbons removal, both BC caused a substantial reduction in PHCs contents in planted as well as non-planted treatments (Fig. 4). Approximately, a loss of 32.0% and 22.0% of initially applied concentrations of PHCs at 1.5% and 3.0%, respectively was observed after 140 d in un-inoculated controls and unplanted treatments. However, the chickpea plant alone phytoremediate approximately 52.0% and 35.0% of initial concentrations of PHCs when grown in 1.5% and 3.0% PHCs, respectively (Fig. 4). While the treatments containing different BC removed higher amounts of PHCs than that of their uninoculated controls (chickpea planted or non-planted treatments but without BC). Approximately, 32.0% and 59.0% PHCs removal (of initial concentrations) was observed in treatments containing BC1 and BC2, respectively in the absence of chickpea plants (at 1.5% PHCs). However, approximately, 72.0% and 76.0% PHCs removal (of initial concentrations) was observed in treatments containing BC1 and BC2, respectively in the planted treatment (at 1.5% PHCs) which is significantly higher as compared to unplanted controls (with BC). Likewise, about 45.0% and 52.0% PHCs removal (of initial concentrations) was observed in treatments containing BC1 and BC2, respectively in the presence of chickpea plants (at 3.0% PHCs) which is significantly higher than unplanted controls at 3.0% PHCs (35.0% for BC1 and 41.0% for BC2).

#### Correlation matrix and principal component analysis

To examine the association or connection between various growth, physiological, antioxidative, and nutritional characteristics of chickpea plants, a correlation analysis was done on mean values of the obtained data (Fig S6). A strong positive correlation among physiological, growth, and nutritional characteristics (i.e., 0.87 to 0.99) was observed when BC was applied in uncontaminated soil. However, a strong negative correlation (i.e., -0.72 to -0.98) was seen between the growth, physiological, and nutrient attributes with antioxidant contents and/or petroleum hydrocarbon contamination. Moreover, a strong positive correlation was noticed between antioxidative contents and hydrocarbon contamination levels. The importance of different treatments with or without the presence of hydrocarbons was presented by PCA. A higher cumulative variation of 97.8% was shown by PC1 (95.2%) and PC2 (2.63%). Overall, a greater variation among applied treatments is illustrated in Fig S7.

## Discussion

Hydrocarbons inclusion in soil reduced the growth, physiology and nutrients assimilation in chickpea plants. Nevertheless, the addition of bacterial consortia (BC1 and BC2) significantly uplifted the harmful impacts of PHCs on the physiological processes and development of chickpea plants grown in PHCs contaminated soil. Seedling emergence was decreased in the present study and the findings were matched with Grifoni et al. [48] who also observed reduced seedling emergence when seeds were sown in oil-spilled soils. The reduced germination may be attributed to the light-weight vaporized fractions of PHCs that permeate the seed coat and cause embryonic death [49]. Moreover, the hydrophobic nature of PHCs hinders the water intake and disturbs the imbibition [50, 51]. However, the inoculation of microbes in PHCs contamination alleviate the pollutant’s toxicity by the possible change in soil pH and C: N ratio [52]. This might be due to exopolysaccharides secretions of bacterial strains that moisten the seed coat and promote early germination [53].

Petroleum hydrocarbons’ existence in the soil leads to stunted plant growth [48] and this was also observed in the present study. Basically, in soil, PHCs minimize the oxygenation by blocking the soil pores and resulting in anaerobiosis conditions for plant roots [54]. The PHCs led to a decrease in root and shoot dry biomass and plant height, as already reported by Ali et al. [37]. The possible cause might be cell injuries that restrict the intake of water and nutrients, and hinder the plant’s growth [5, 54]. The phytotoxic effects of PHCs negatively altered plant biochemical processes by changing cell membrane permeability, water potential and enzymatic disruptions (that cause the production of ROS, H_2_O_2_ and ethylene), which subsequently affect plants’ metabolic pathways [55, 56]. The incorporation of mixed bacterial strains in this study resulted in an increased plant growth by mitigating the harmful effects of PHCs on plants. The microbes used in current study have already been identified as plant growth promoters and their details are already provided in our previous work [36]. In both cultures, the presence of mixed microbes which are belonging to *Achromobacter*, *Alcaligenes*, *Sphingobacterium* and *Stenotrophomonas* possess the capability to produce growth hormones such as auxin, gibberellins, ACC deaminase, iron and phosphate solubilizing enzymes and oxidase to convert H_2_O_2_ to H_2_O which strengthen the plants against negative impacts of PHCs stress as it was also shown by other studies [21, 57].

Plants physiology is also greatly influenced by hydrocarbons toxicity which lowers the levels of chlorophyll pigments and carotenoid contents in leaves [58]. In stressed condition, the chloroplast absorb fewer photons from sun to accelerate the ETR to absorb the carrying energy [59]. This may be because of the elevated H_2_O_2_ levels, that damage the chloroplast membrane and thylakoid structure under stressed conditions [60]. In case of the addition of bacterial consortia, the harmful impacts of PHCs on photosynthetic processes and chlorophyll synthesis has substantially been reduced [61]. However, the inclusion of microbes halted the synthesis of H_2_O_2_ and boosted the photosynthetic activities and the chlorophyll synthesis probably by lowering oxidative damage [62]. In the present study, the alteration in cell membrane structure might be due to the volatilized fractions of PHCs that result in cell ruptures and affect the MSI and RWC of leaves [10]. The PHCs also disturb the photosynthetic rates by reducing CO_2_ and H_2_O assimilation, the prevailing conditions initiate ROS generation, which disturbs the plant’s physiology [63].

The addition of both bacterial consortia in contaminated soil reduces the PHCs toxicity and strengthens the plants’ physiology. Under stress, the microbes produce ACC-deaminase that hydrolyze the ACC and minimizes the ethylene production in plants [64, 65]. In the present study, both cultures performed well in minimizing the negative impacts of PHCs on the physiology, growth and development of plants grown under PHCs stress, these results favorably support the findings of Ali et al. [8] and Farooq et al. [66].

In the current work, the proline and other antioxidant levels were also found higher in leaves that indicate the progress of stress related enzymes in PHCs contamination. According to Hasanuzzaman et al. [67], the proline is involved in maintaining cell structures, breaking down of free radicals, and stimulating the stress related intracellular metabolic processes. Therefore, the enhanced proline builds up in leaf tissues allowed stressed plants to balance their redox potential and control cellular metabolites levels [68]. The oxidative stress in plants causes cell membrane damage and disrupt ionic homeostasis, which initiates the production of higher amounts of ROS in tissues [6]. In general, proline mitigates these effects via lowering stress, regulating mitochondrial activity, balancing cellular division, strengthening membrane stability, and preventing electrolyte leakage from plant cells [69, 70].

This research found that stress-responsive enzymes like SOD and POD and CAT were highly active under PHCs stress. The antioxidative system protects plants against the damaging effects of contaminants [71]. The SOD serves as the plant’s first line of defense against ROS created stress [72]. Superoxide dismutase initially converts superoxide to H_2_O_2_ and O_2_, then POD and CAT convert this H_2_O_2_ to water and oxygen [73]. Plants produce antioxidant enzymes that help plants against abiotic stresses [74]. Here, it is found that the activity of POD, SOD, and CAT is changed when bacterial consortia were added in hydrocarbons contaminated treatments. Microbes, upregulate SOD activity and create melanin, a hormone used for scavenging free radicals [75]. The chemical pathways for protein synthesis begin right after the recovery of superoxide by SOD activity [43]. In the current study, the bacterial consortia (BC1 and BC2) performed well in PHCs stress, reducing the detrimental impacts of PHCs on plants via improving the stress responsive enzymes.

In this study, PHCs reduced nutrient assimilation in chickpea plants. A change in soil pH and increased C: N ratio might be a possible reason behind lower N intake, as it was already presented by Xu et al. [76]. Thus, N deficiency could subsequently affect the cellular enlargement and leaf growth by disturbing the amino acid formation and protein synthesis. In the similar way, P and K intake in plants was also decreased in PHCs stress [21]. Since, the P and K control several metabolic processes including respiration, energy transfer, signal transduction, macro-molecules biosynthesis and photosynthesis, which are essentially required for plant growth [77]. Though, the addition of bacterial consortia could significantly improve the nutrient uptake by plants probably due to secretions of stress reliever enzymes and/or breakdown of PHCs [15].

In present study the PHCs removal was slower in earlier phase of incubation; however, a substantial increase in microbial growth and PHCs degradation was noticed with time. This could be explained by the slow lag phase of microbial growth as observed by Omrani et al. [78]. In short, the indigenous microbial communities, present at sites 1 and site 5 were able to remediate a substantial amount of PHCs in liquid cultures. While other sites have relatively lesser potential to degrade PHCs. The considerable differences in the removal of PHCs by the microbes from the different sites could be due to the aging of PHCs and/or geographical effects [79]. Because it has been observed that microbial communities from different origins have different potentials for hydrocarbon degradation [79].

Microbes utilize PHCs as a source of carbon and energy, which was evident from the increase in OD_600_ values of culture after incubation [80]. According to reports, microbes have a special ability to use hydrocarbons via various enzymes and break them down into smaller and/or non-toxic substances [81]. Microbial growth, estimated in terms of OD_600_, showed a significant increase with the removal of PHCs during the incubation period, indicating the growth potential of enriched microbes under PHCs contamination [26]. In lateral stages, after three sequential transfers PHCs removal was further increased, the enhanced removal of PHCs could be due to the improved adaptation and selective enrichment of PHCs degraders [82]. Overall, the contaminated sites harbor microorganisms that are capable of degrading hydrocarbons in liquid culture. These microbes (bacteria) were isolated from the enriched culture and mixed to construct bacterial consortia which were used in the rhizoremediation experiments.

Various rhizoremediation experiments describe the importance of microbial communities (due to diversified enzymatic activities and adaptability) for better plant growth and PHCs remediation from polluted environments [5, 28, 81]. The developed BC (in the present work) have the ability to remove a significant amount of hydrocarbons from soil in relatively short exposure time. The dual nature of selected microbes (to promote PHCs degradation and plant growth) remarkably improved the rhizo-degradation process. Moreover, plants can tolerate higher amounts of pollutants in the rhizosphere and can potentially phytoremediate a specific concentration of PHCs contaminants from the soil [83].

Furthermore, the rhizoremediation was highest in the presence of added microbes. Increased PHCs removal from the soil might be possible due to the secretions of organic acids, protonation, release of chelators (chelation), chemical transformation, and phosphate mineralization by inoculated bacteria [12]. This in turn increases the bioavailability of PHCs to plants and microbes [8]. Here, in current work, the selected bacterial strains from genera; *Achromobacter*, *Alcaligenes*, *Sphingobacterium*, and *Stenotrophomonas* were used to construct two different bacterial consortia. The *Alclaligene* sp. secretes phosphatase enzymes to mineralize the fixed-P from soil [84] and improves the rhizoremediation capability by enhancing plant growth. The *Achromobacter denitrificans* sp. are capable of degrading aromatic and long chain PHCs by the production of hexadecane monooxygenase and 2,3-dioxygenase [85]. Arulazhagan et al. [81, 86] reported that the *Stenotrophomonas maltophilia* AJH1 can degrade volatile PHCs. Furthermore, the *Sphingobacterium* sp. KM-02, is also known for its biodegradation potential for volatile fractions of PHCs [87]. Additionally, the *Sphingobacterium* ability to produce biosurfactants may enhance the bioavailability of PHCs and thus can accelerate the remediation process [88].

Overall, hydrocarbon presence in soil exerted acute toxicity in plants, while the addition of two different BC not only improved the plant growth but also enhanced the nutrient uptake for strengthening the plant’s physiology. These microbes due to their diversified enzymatic properties removed a substantial amount of PHCs from soil and in that way reduced the risk of phytotoxicity in plants. In general, the BC2 showed better growth and hydrocarbons degradation rather than BC1. So, the native BC could be used for plant growth promotion and rhizoremediation purposes in soil contaminated with organic pollutants including PHCs.

## Conclusions

The purpose of this research was to evaluate and compare the efficacy of two constructed bacterial consortia in reducing hydrocarbon stress and boosting chickpea plants in hydrocarbon-contaminated soil. The presence of PHCs inhibited the development of chickpea plants. More importantly, the microbes reduced the phytotoxic impacts of PHCs on chickpea growth and thus enhanced plant growth, most likely through antioxidative defense and plant growth-promoting properties. Additionally, the application of bacterial consortia with attributes that promote plant growth significantly boosted the removal of PHCs from soil and enhanced the capability of chickpea plants to eliminate PHCs. Largely, both bacterial consortia were quite successful in their employment; however, BC2 performed better than BC1 in promoting plant growth and in degrading higher amount of PHCs. Based on our results, the unique bacterial consortia may be a promising contender for future phytoremediation efforts due to their ability to aid in PHCs degradation and boost plant growth in PHCs contaminated soil.

## Electronic supplementary material

Below is the link to the electronic supplementary material.

Supplementary Material 1

## Data Availability

The original contributions presented in this study are included in the article and/or supplementary material, further inquiries can be directed to the corresponding author.
