# Supplementary material for: Improved chickpea growth, physiology, nutrient assimilation and rhizoremediation of hydrocarbons by bacterial consortia
Source: BMC Plant Biol. 2024 Oct 19;24:984. doi: 10.1186/s12870-024-05709-x (PMC11490054; doi:10.1186/s12870-024-05709-x)
Supplement: Supplementary file 1 — Supplementary Material 1 [file 12870_2024_5709_MOESM1_ESM.docx]

**Supplementary material**

**Improved chickpea growth, physiology, nutrient assimilation and rhizoremediation of hydrocarbons by bacterial consortia**

Muhammad Hayder Ali^1^, Muhammad Imran Khan^1,^*, Fiza Amjad^1^, Naeem Khan^2^, Mahmoud F. Seleiman^3^

*^1^Institute of Soil and Environmental Sciences, University of Agriculture, Faisalabad, 38000, Pakistan*

*^2^Agronomy Department, Institute of Food and Agricultural Sciences, University of Florida, Gainesville FL, 32611, USA*

*^3^ Plant Production Department, College of Food and Agriculture Sciences, King Saud University, P.O. Box 2460, Riyadh 11451, Saudi Arabia*

*Corresponding author: Muhammad Imran Khan

Email: [khanimran1173@yahoo.com](mailto:khanimran1173@yahoo.com)

**Table S1** Treatments detail of rhizoremediation experiment.

| **Treatments** | | **Detail** |
| --- | --- | --- |
| Control | Uncontaminated soil | Uncontaminated soil with plants |
|  | 1.5% PHCs contamination without plant | 1.5% petroleum hydrocarbons contaminated soil |
|  | 1.5% PHCs contamination | 1.5% petroleum hydrocarbons contaminated soil with plants |
|  | 3.0% PHCs contamination without plant | 3.0% petroleum hydrocarbons contaminated soil |
|  | 3.0% PHCs contamination | 3.0% petroleum hydrocarbons contaminated soil with plants |
| BC1 | Uncontaminated soil | Uncontaminated soil with plants and BC1 |
|  | 1.5% PHCs contamination without plant | 1.5% petroleum hydrocarbons contaminated soil with BC1 |
|  | 1.5% PHCs contamination | 1.5% petroleum hydrocarbons contaminated soil with plants and BC1 |
|  | 3.0% PHCs contamination without plant | 3.0% petroleum hydrocarbons contaminated soil |
|  | 3.0% PHCs contamination | 3.0% petroleum hydrocarbons contaminated soil with plants and BC1 |
| BC2 | Uncontaminated soil | Uncontaminated soil with plants and BC2 |
|  | 1.5% PHCs contamination without plant | 1.5% petroleum hydrocarbons contaminated soil with BC2 |
|  | 1.5% PHCs contamination | 1.5% petroleum hydrocarbons contaminated soil with plants and BC2 |
|  | 3.0% PHCs contamination without plant | 3.0% petroleum hydrocarbons contaminated soil |
|  | 3.0% PHCs contamination | 3.0% petroleum hydrocarbons contaminated soil with plants and BC2 |

BC1, bacterial consortium 1 of selected bacterial strains *A. faecalis*, *Alcaligenes* sp., *A*. *denitrificans* and *S*. *spiritivorum*; BC2, bacterial consortium 2 of selected bacterial strains *S*. *spiritivorum*, *A*. *xylosoxidans*, *Stenotrophomonas* sp., *A. faecalis* and *S*. *rhizophila*; PHCs, petroleum hydrocarbons.


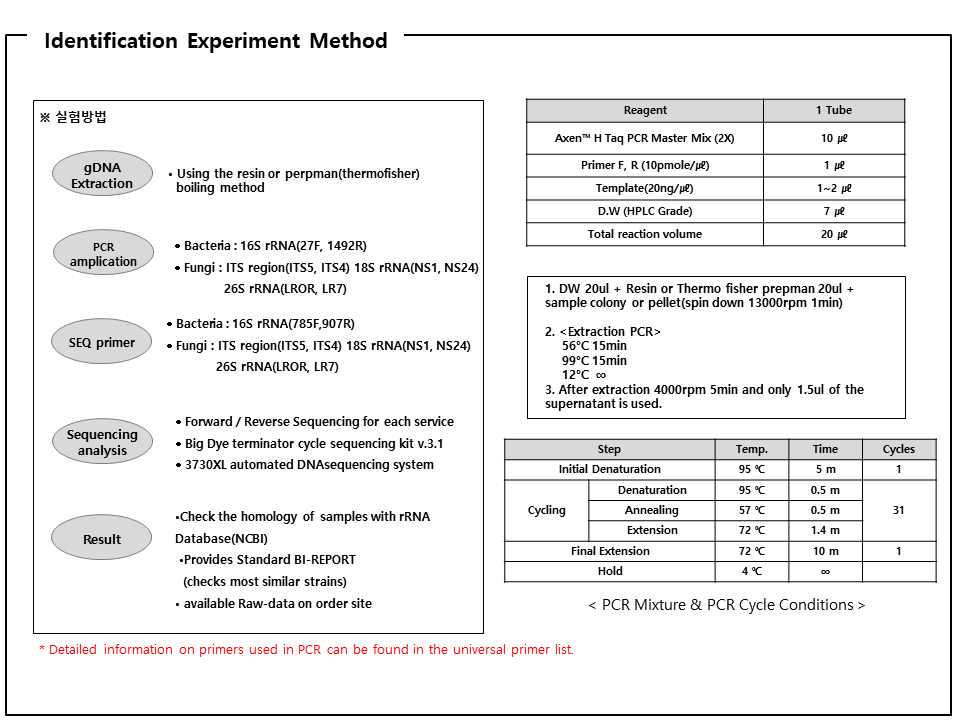


**Fig. S1** DNA extraction, primer information, and PCR conditions used by Macrogen, Inc, South Korea for sequencing of selected bacterial isolates in this study. The universal primers (V3-V4: Bakt_341F: CCTACGGGNGGCWGCAG and Bakt_805R: GACTACHVGGGTATCTAATCC) were used by Macrogen for identification of bacterial strains.

**Fig. S2** PHCs degradation during the first enrichment (A), first subculturing (B) and second subculturing (C) experiments with soil collected from five different contaminated sites. Each column represents the mean value of treatment, and each bar represents the standard error within the same treatment. The small alphabetical letters present the statistical variation among different means at the same time point. Site 1, soil collected from PHCs contaminated site in Abbottabad; Site 2, soil collected from PHCs contaminated site in Rawalpindi, Site 3; soil collected from PHCs contaminated site in Faisalabad; Site 4, soil collected from PHCs contaminated site in Bahawalpur; Site 5, Soil collected from PHCs contaminated site in Muzaffargarh.

**Fig. S3** Microbial growth (estimated) during the 1^st^ (A), and 2^nd^ (B) subculturing experiments with soil collected from five different contaminated sites. The microbial growth was not measured in first enrichment experiment due to the presence of soil particles which affects its OD value. *OD*, optical density at 600nm. The alphabetical letters present the statistical variation among different means. PHCs, Petroleum hydrocarbons contaminated sites; Site 1, soil collected from PHCs contaminated site in Abbottabad; Site 2, soil collected from PHCs contaminated site in Rawalpindi, Site 3; soil collected from PHCs contaminated site in Faisalabad; Site 4, soil collected from PHCs contaminated site in Bahawalpur; Site 5, Soil collected from PHCs contaminated site in Muzaffargarh.

**Fig. S4** Effect of different levels of pH on PHCs removal (A and C) and microbial growth (B and D) by cultures enriched from site 1 (A and B) and site 5 (C and D). The alphabetical letters present the statistical variation among different means. PHCs, petroleum hydrocarbons; Site 1, soil collected from PHCs contaminated site in Abbottabad; Site 5, Soil collected from PHCs contaminated site in Muzaffargarh.

**Fig. S5** Effect of different temperatures on PHCs removal (A and C) and microbial growth (B and D) by cultures enriched from site 1 (A and B) and site 5 (C and D). The alphabetical letters present the statistical variation among different means. PHCs, petroleum hydrocarbons; Site 1, soil collected from PHCs contaminated site in Abbottabad; Site 5, Soil collected from PHCs contaminated site in Muzaffargarh.


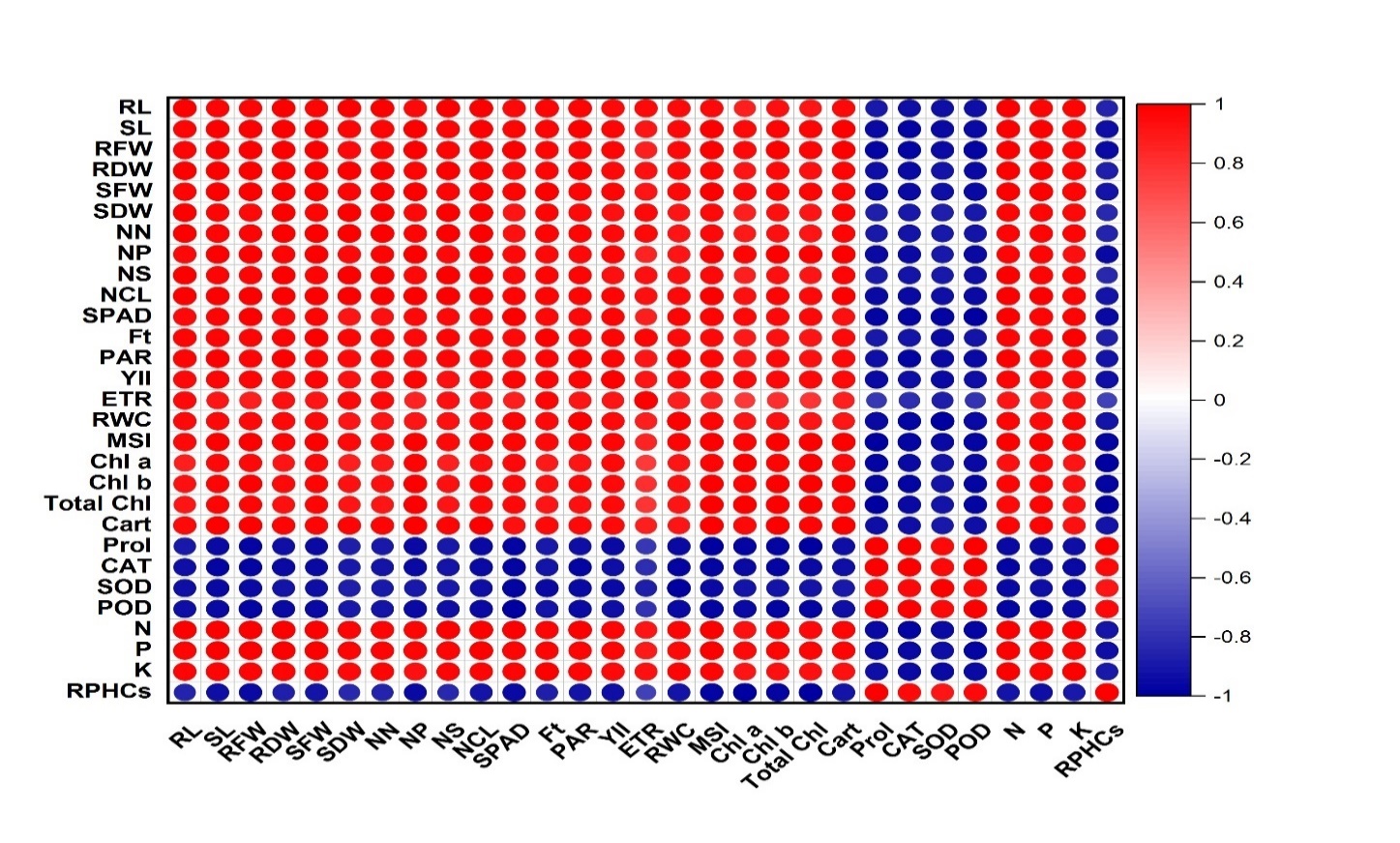


**Fig. S6** The correlation plot represents a correlation matrix among different growth, physiological, nutrients, and antioxidative attributes of chickpea plants concerning petroleum hydrocarbons in soil. The size of the square shows the strength of the relationship (high, moderate or low) of different attributes of the chickpea crop. The dark red and dark blue color shows a highly positive or negative correlation, respectively. The color legend present on the right side of the correlation plot shows the corresponding colors and the correlation coefficient. RPHCs; residual petroleum hydrocarbons; K, potassium; P, phosphorus; N, nitrogen; POD, peroxidase; SOD, superoxide dismutase; CAT, catalase; Prol, proline contents; Cart, carotenoids, Total Chl, total chlorophyll; Chl b; chlorophyll b; Chl a, chlorophyll a; MSI, membrane stability index; RWC, relative water contents; ETR, electron transport rate; YII, quantum yield; PAR, photosynthetically active radiation; SPAD, Chlorophyll value; Ft, fluorescence yield; NG, number of grains, NSs, number of spikelets; NS, number of spikes; GWt, grains weight; SDW, shoot dry weight; SFW, shoot fresh weight; RDW; root dry weight; RFW, root fresh weight; SpL, spike length; SL, shoot length; RL, root length.


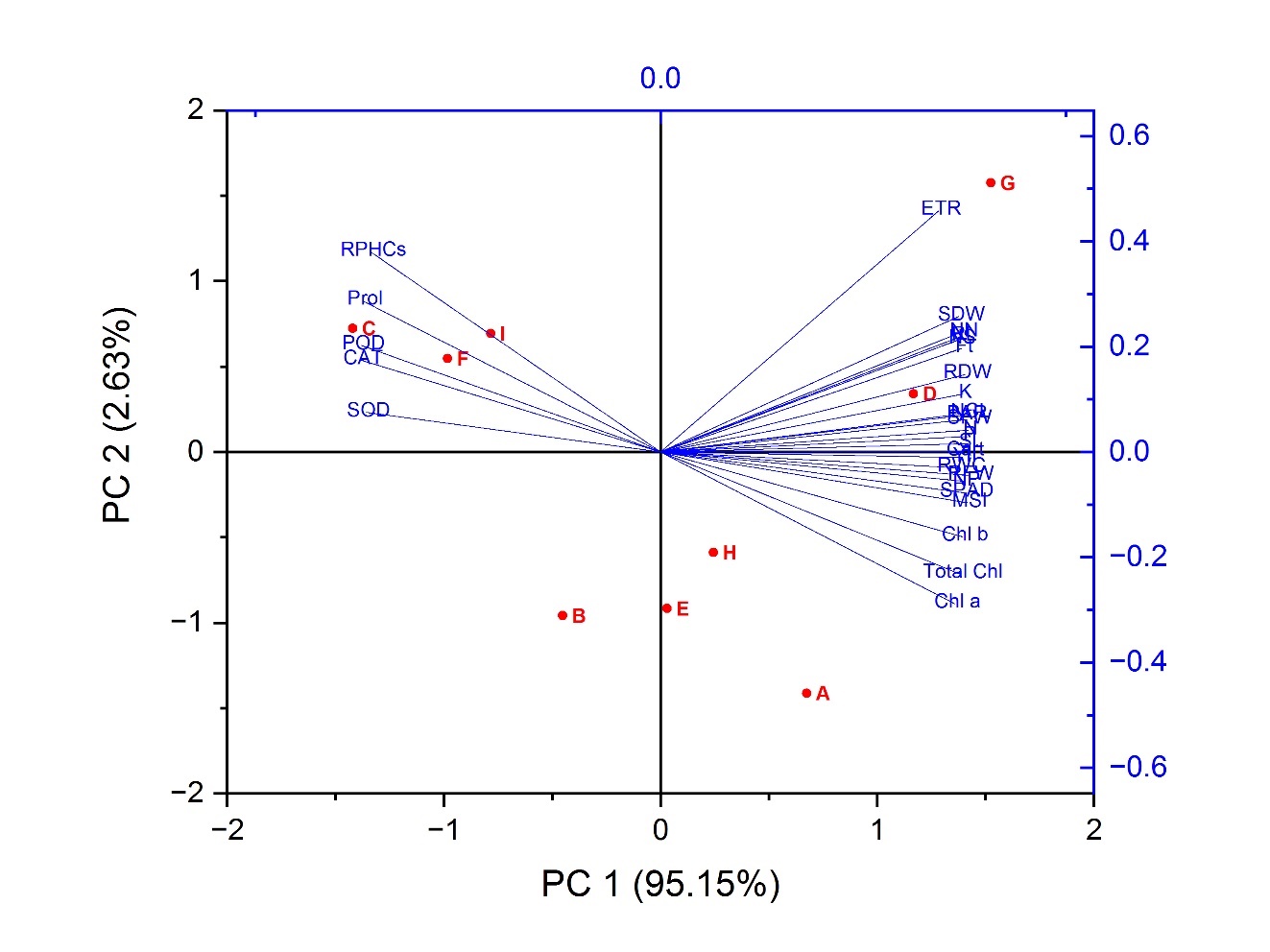


**Fig. S7** Principal component analysis plot of various growth, physiological, nutrients, and antioxidative attributes of chickpea plants concerning petroleum hydrocarbons in soil. PC1 denotes the PHCs contents which explains the majority of differences. The different alphabets denote the different treatments, A; uncontaminated soil, B; 1.5% PHCs contamination, C; 3.0% PHCs contamination, D; uncontaminated soil + MC1, E; 1.5% PHCs contamination + MC1, F; 3.0% PHCs contamination + BC1, G; uncontaminated soil + BC2, H; 1.5% PHCs contamination + MC2, I; 3.0% 37PHCs contamination + MC2, scattered all over the plot. Each parameter is represented by an arrow, and arrow length approximates their difference and the angle between different parameters shows the extent of correlation. RPHCs; residual petroleum hydrocarbons; K, potassium; P, phosphorus; N, nitrogen; POD, peroxidase; SOD, superoxide dismutase; CAT, catalase; Prol, proline contents; Cart, carotenoids, Total Chl, total chlorophyll; Chl b; chlorophyll b; Chl a, chlorophyll a; MSI, membrane stability index; RWC, relative water contents; ETR, electron transport rate; YII, quantum yield; PAR, photosynthetically active radiation; SPAD, Chlorophyll value; Ft, fluorescence yield; NG, number of grains, NSs, number of spikelets; NS, number of spikes; GWt, grains weight; SDW, shoot dry weight; SFW, shoot fresh weight; RDW; root dry weight; RFW, root fresh weight; SpL, spike length; SL, shoot length; RL, root length.
